# High-Stretchable and Transparent Ultraviolet-Curable Elastomer

**DOI:** 10.3390/polym16243464

**Published:** 2024-12-11

**Authors:** Lei Chen, Yongchang He, Lu Dai, Wang Zhang, Hao Wang, Peng Liu

**Affiliations:** 1 College of Mechanical and Vehicle Engineering, Hunan University, Changsha 410082, China; 2 Engineering Product Development, Singapore University of Technology and Design, Singapore 487372, Singapore; 3 School of Mechanical Engineering, Hunan University of Science and Technology, Xiangtan 411201, China

**Keywords:** elastomer, stretchability, transparency, stretchable sensor, 3D printing

## Abstract

This work introduces an ultraviolet (UV)-curable elastomer through the co-polymerization of aliphatic polyurethane acrylate and hydroxypropyl acrylate via UV irradiation. The UV-curable elastomer presents superior mechanical properties (elongation at a break of 2992%) and high transparency (94.8% at 550 nm in the visible light region). A robust hydrogel–elastomer stretchable sensor is fabricated by coating an ionic hydrogel on the surface of an elastomer, which enables real-time monitoring of human motion. In addition, the UV-curable elastomer can be used for 3D printing, as demonstrated by complex lattice structures using a digital light processing 3D printer.

## 1. Introduction

Flexible electronics, due to their lightweight, stretchability, and electrical performance, are currently used in various applications, including e-skin [1], soft robotics [2], [3], and human–machine interfaces [4,5,6], etc. [7,8,9,10,11]. A typical flexible electronic device consists of a substrate, backplane electronics, a frontplane, and encapsulation [12]. Each component must tolerate a certain degree of deformation to remain flexible, without compromising functionality. For flexible electronic systems, the performance of substrates plays a crucial role. The substrates should be able to bend and stretch without breaking while maintaining the electrical properties for the integration of electronics [13].

In recent years, several polymer substrates have been explored for their potential use in flexible electronic systems, including polyimide (PI) [14], polyethylene terephthalate (PET) [15], silicone rubber (typically PDMS) [16], and UV-curable elastomers [17]. Of these substrates, elastomer-based materials such as silicone rubber and UV-curable elastomers offer several advantages over PI and PET, including high stretchability and conformability to human skin [18]. For example, the elongation at break for PI and PET is only 3% and 12%, respectively, while elastomer-based materials provide much higher stretchability with elongation exceeding 100% [19]. In addition, compared to silicone rubber, UV-curable elastomers cure much more rapidly, within seconds at room temperature and under milder reaction conditions [20]. A typical UV-curable elastomer consists of a mixture of monomers, oligomers, and photoinitiators [21]. When exposed to UV radiation, the photoinitiator molecules absorb the energy and undergo a chemical reaction, initiating cross-linking between the monomers and oligomers [21,22]. This cross-linking forms a three-dimensional network of long, soft polymer chains, allowing the elastomer to stretch when force is applied and return to its original state when the force is removed. Several UV-curable elastomers have been developed for application in soft actuators/robots [23], stretchable electronic sensors [24], etc. However, UV-curable elastomers currently suffer from low elongation at break and limited transparency, which can hinder their practical applications.

Herein, we selected aliphatic polyurethane acrylate (PUA) as oligomer, hydroxypropyl acrylate (HPA) as monomer, poly(ethylene glycol) diacrylate (PEGDA) as cross-linker, and ethyl (2,4,6-trimethylbenzoyl) phenylphosphinate (TPO-L) as photoinitiator to prepare liquid resin for UV-curable elastomer. The resulting elastomer exhibits superior performance, including superior mechanical properties (elongation at a break of 2992%) and high transparency (94.8% at 550 nm). We further demonstrate the fabrication of a hydrogel–elastomer stretchable sensor with high sensitivity by coating a layer of ionic hydrogel onto the surface of the elastomer substrate, which can be used for real-time monitoring of human motion. In addition, we demonstrate the capacity of 3D printing of complex structures using this elastomer.

## 2. Experimental Section

### 2.1. Materials

The materials for UV-curable elastomers included PUA, HPA, PEGDA, and TPO-L. PUA (RJ425), PEGDA, and TPO-L were purchased from RYOJI Chemical Co., Ltd., Guangzhou, China, via Taobao. HPA was obtained from Chengdu Guangju Technology Co., Ltd., Chengdu, China. TPO-L was chosen for its high absorption in the deep blue to near UV range [5]. All materials were commercially obtained and used without any further purification.

### 2.2. Preparation of UV-Curable Liquid Resin

To prepare the liquid resin of elastomer, PUA, HPA, and PEGDA were mixed in different weight ratios and stirred mechanically for 1 h until the resin was uniform. TPO-L, a free radical photoinitiator, at a rate of 3.0 wt. %, was added into the mixture to enable the initiation of the polymerization process during exposure to UV light. The resulting liquid resin mixture was used as a precursor for the fabrication of elastomer. The liquid resin mixture with different ratios of PUA and HPA and different PEGDA cross-linkers are shown in Table 1 and Table 2.

### 2.3. Fabrication of UV-Curable Elastomer

The liquid resin mixture was poured into a 3D printed mold using standard resin from Anycubic Co., Ltd (Shenzhen, China) and then exposed to UV light at a wavelength of 405 nm (ZLUVLAMP, via Taobao, Shenzhen, China). The intensity of UV light was 610 mW/cm^2^. After a curing time of approximately 20 s, the cured elastomer was detached from the mold and subjected to further tests or applications as required.

### 2.4. Fabrication of Hydrogel–Elastomer Stretchable Sensor

The cured elastomer was immersed in an ionic hydrogel precursor solution for 10 min and cured using UV light of 405 nm for 30 s. Alternatively, the ionic hydrogel precursor solution was directly cured on the surface of the elastomer using a patterned UV light of 405 nm. The uncured hydrogel was then removed using water or ethanol and dried with nitrogen gas.

### 2.5. Characterization

The tensile tests were conducted on a mechanical testing apparatus (ZQ 990A, Dongguan Zhiqu Precision Instrument Co., Ltd., Dongguan, China) at room temperature with a sensor of 200 N. The transmittance of the cured elastomer was measured using a UV-vis spectrophotometer (NOVA-EA, Shanghai Fuxiang Optical Co., Ltd., Shanghai, China) over a range from 400 nm to 800 nm. The performance of the hydrogel–elastomer stretchable sensor was tested using a digital meter (Keithley 2611B, Keithley Instruments, purchase location: Shanghai, China). The two ends of the sensor were connected to the electrodes of the source meter, and the tests were conducted under a stable voltage. The resistance of the sensor was monitored and recorded over time. The relative change in resistance was calculated using the following formula: ΔR/R_0_ = (R − R_0_)/R_0_, where R_0_ and R represent the resistance without and with applied strain, respectively [5].

## 3. Results and Discussion

### 3.1. Preparation of UV-Curable Elastomer

**Figure 1:**
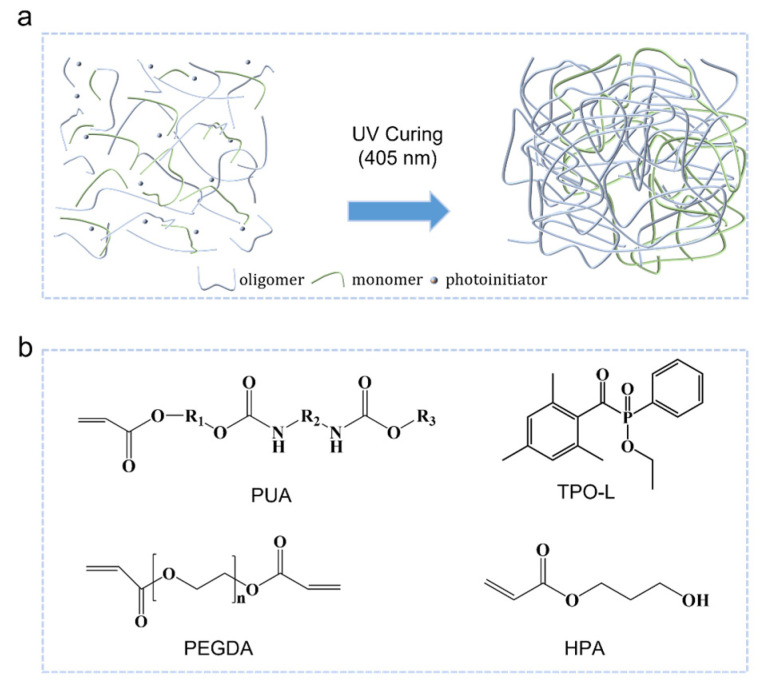
Preparation of UV-curable elastomer. (**a**) Schematic of elastomer formed by covalent cross-linking of oligomer and monomer. (**b**) The chemical structures of PUA, HPA, PEGDA, and TPO-L. HPA may have multiple isomers, one of the formulas is shown here.

The UV-curable liquid resin was prepared by mixing oligomer and monomer consisting of PUA, HPA, and PEGDA. The elastomer composition contains 3 wt. % of TPO-L as a photoinitiator, which is suitable for UV-curing at a wavelength of 405 nm. The chemical structures of PUA, HPA, PEGDA, and TPO-L are shown in Figure 1a,b. When exposed to UV light, the TPO-L photoinitiator absorbs energy and converts to an active group, generating free radicals that initiate the polymerization of PUA and HPA. PUA is a mono-acrylate-based oligomer with hydroxyethyl alkyl acrylate and a soft aliphatic group. R_1_ refers to hydroxyethyl alkyl acrylate, while the others represent diisocyanate and aliphatic groups. HPA is a mono-acrylate-based monomer with soft segments. These soft segments form extended linear chains that entangled with each other after co-polymerization, resulting in an elastic network. PEGDA is a diacrylate-based cross-linker that can improve the cross-linking density. However, a cross-linking agent is not always required during the UV-curing process.

### 3.2. Transparency of Elastomer

**Figure 2:**
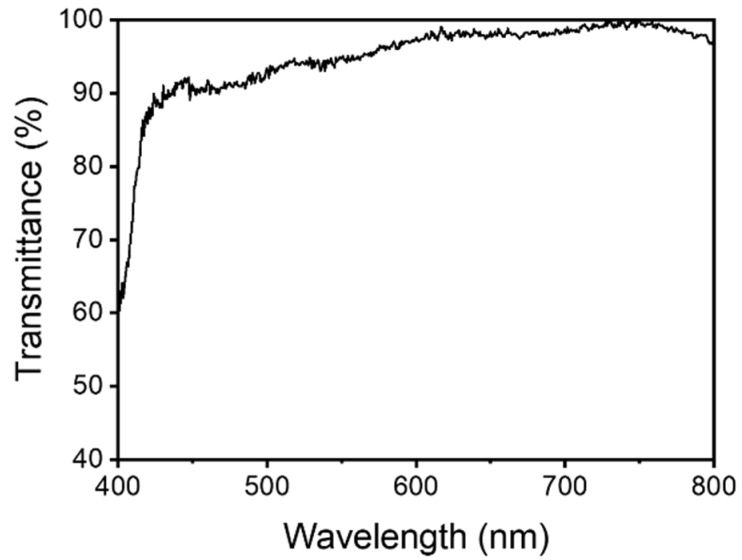
Transparency of UV-curable elastomer. The transmittance spectrum of the as-fabricated UVE-A3 elastomer in the visible light region.

The as-fabricated elastomer exhibits excellent optical transmittance in the visible range. Figure 2 shows the transmittance spectrum of a UVE-A3 sample over a wavelength range from 400 to 800 nm. The thickness of the sample is 0.1 mm. The results indicate that the elastomer shows high transmittance across the entire visible light spectrum. Notably, the sample achieves a transmittance of 94.8% at 550 nm. However, a decrease in transmittance is observed near 400 nm, which is attributed to the absorption of the UV initiator TPO-L.

### 3.3. Mechanical Properties of Elastomer

**Figure 3:**
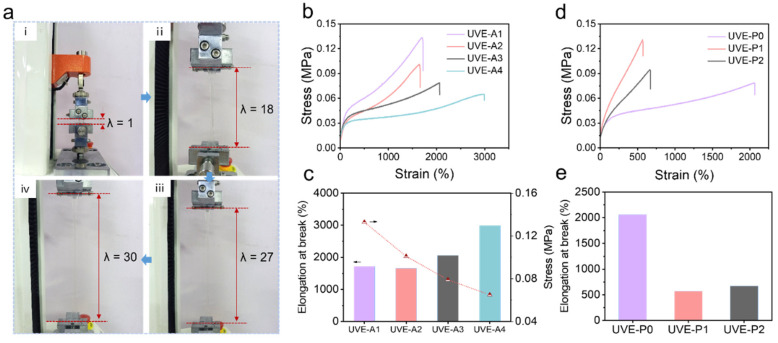
Mechanical properties of elastomer. (**a**) Snapshots of stretching a transparent elastomer specimen by about 30 times. (**i**) The original length is defined as λ. Stretching times: (**ii**) 18 times, (**iii**) 27 times, (**iv**) 30 times. (**b**) Stress–strain curves of as-fabricated elastomer with different weight ratios of PUA and HPA. (**c**) The obtained elongation at break and tensile stress from (**b**). (**d**) Stress–strain curves of as-fabricated elastomer with different PEGDA cross-linkers. (**e**) The obtained elongation at break from (**d**).

Uniaxial tensile tests were performed to investigate the mechanical properties of the elastomer. Figure 3a demonstrates the mechanically stretchable properties of the as-fabricated elastomer, which can be stretched up to about 30 times its original length. The stress–strain curves of the as-fabricated elastomers with varying ratios of PUA oligomer and HPA monomer (Figure 3b) were obtained. The results indicate that UVE-A1, UVE-A2, UVE-A3, and UVE-A4 exhibit elongation at break values of 1721%, 1657%, 2064%, and 2992%, respectively (Figure 3c). The elongation at break increases with increasing HPA monomer content, and UVE-A4 demonstrates the best mechanical properties with the highest elongation at break of 2992%. The excellent flexibility of the elastomer is attributed to the soft segments of photopolymerized HPA. In addition, UVE-A1 exhibits the highest tensile strength of 0.13 MPa due to the hard segments from the PUA monomer. During the UV photopolymerization process, HPA monomer forms linear chains, which extend the length of the linear chains within the PUA network. This increase in chain length enhances the stretchability of the network system. However, the addition of HPA monomer results in a reduction in stiffness, leading to a decrease in tensile strength. In Figure 3d, the strain–stress curves of the as-fabricated elastomers with different PEGDA cross-linkers are presented. The results show that the PEGDA cross-linker leads to an increase in tensile strength while decreasing the elongation at break (Figure 3e). This phenomenon is attributed to the addition of PEGDA into the elastomer chains, which increases the cross-linking density of the PUA and HPA network, resulting in high tensile strength but low elongation of the elastomer.

### 3.4. Hydrogel–Elastomer Stretchable Sensor

**Figure 4:**
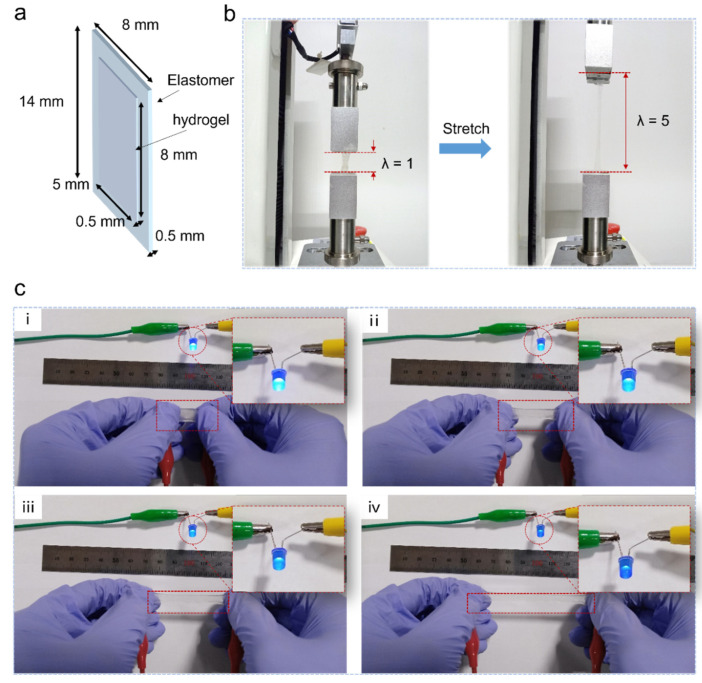
Fabrication of hydrogel–elastomer stretchable sensor. (**a**) The combination of elastomer and ionic conductive hydrogel. (**b**) The sensor under large deformation (e.g., stretch) without debonding. (**c**) The LED bulb in the circuit with the sensor. (**i**–**iv**) The LED brightness decreases with increased sensor stretching.

The elastomer exhibits high transparency and superior mechanical properties compared to previously reported elastomers (Table 3), making it a promising substrate for transparent, stretchable sensors. Currently, most transparent, stretchable sensors utilize thin graphene, metal grids, and PEDOT: PSS as conductors [7]. However, repeated stretching of these devices can lead to material failure due to the breakage of the interface between the rigid and flexible components. Ionic conductive hydrogels have recently been reported as transparent conductors for their high stretchability and conductivity to form a more stable conductor-substrate interface [5]. Based on this, we developed a transparent, stretchable sensor by introducing a hydrogel–elastomer interface through in-situ fabrication technology. This process involved immersing the as-fabricated elastomer in the ionic hydrogel precursor solution for approximately 10 min or coating it onto one side of the elastomer, followed by curing under 405 nm UV light for 30 s. The ionic hydrogel precursor solution was prepared according to previous work [5]. The thickness of both the hydrogel film and the elastomer is 0.5 mm. As shown in Figure 4a,b, the hydrogel was coated onto the elastomer and the resulting hydrogel–elastomer sensor was able to withstand a 500% stretch without debonding. These results demonstrate the robust interfacial stability between the hydrogel and elastomer. Figure 4c shows the hydrogel–elastomer stretchable sensor connected with a direct-current (DC) voltage and a light-emitting diode (LED) bulb by two wires. The intensity of the LED varies with the applied strain. This is because, as the hydrogel-coated elastomer stretchable sensor is stretched, the electrical resistance of the sensor increases, leading to a corresponding decrease in the brightness of the LED. The results indicate that the combination of elastomer and ionic conductive hydrogel has great potential for the development of stretchable strain sensors and other electronic devices.

### 3.5. Human Motion Detection

**Figure 5:**
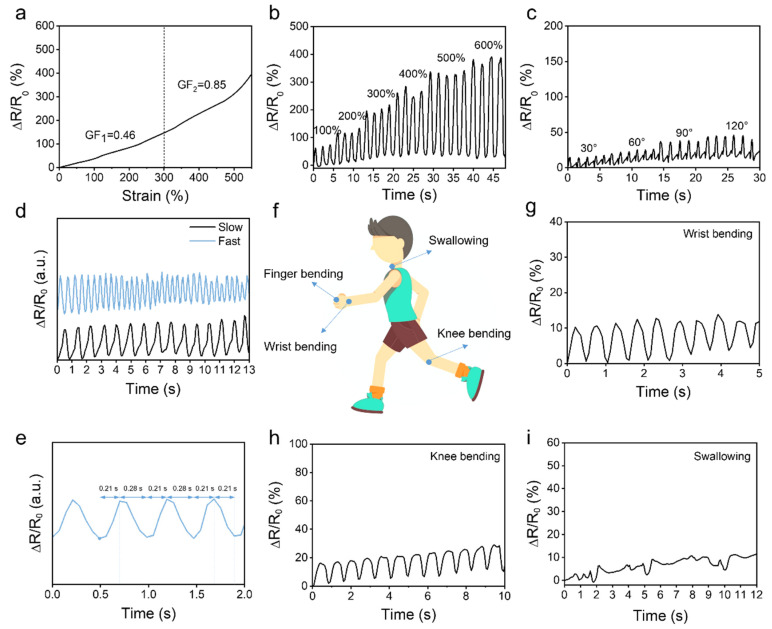
The performance of the hydrogel–elastomer stretchable sensor. (**a**) The relationship between ΔR/R_0_ and the strain of the sensor. (**b**) The resistance response of the sensor to increasing tensile strains. (**c**) The relative resistance changes in the sensor attached to the finger upon different bending angles and (**d**) upon different bending speeds. (**e**) The response and recovery time of the sensor. (**f**–**i**) The relative resistance changes in the sensor for human body movements, such as wrist bending, knee bending, and swallowing.

Hydrogel–elastomer stretchable sensor has emerged as a promising flexible electronic device with a wide range of potential applications, particularly in the field of human motion detection. The sensitivity of the sensor was tested. The characterization details of the sensor are shown in the experimental section. We defined two sensitivity strain ranges: 0–300% and 300–550%. As shown in Figure 5a, the ΔR/R_0_ value of a hydrogel–elastomer stretchable sensor gradually increases as the strain is increased from 0% to 550%. By linearly fitting the relationship between strain and ΔR/R_0_, the gauge factor (GF) can be obtained. The GF is 0.46 in the strain ranging from 0% to 300%, and a higher GF of 0.85 is obtained in the strain ranging from 300% to 550%. Figure 5b shows the relative resistance changes in hydrogel–elastomer stretchable sensors under different strains ranging from 100% to 600%. The resistance changes increase as the strain increases, but the strain sensor remains functional even under a large strain of 600%.

The hydrogel–elastomer stretchable sensor was conformally attached to various positions on a volunteer’s body to monitor the motions in real time. When the sensor was fixed on the volunteer’s finger, the resistance of the sensor increased to different levels upon increasing deformation caused by bending the finger at angles of 30, 60, 90, and 120° (Figure 5c). In addition, we tested the resistance change value of the finger at different bending speeds at 90° and found that the response frequency increased when the finger was bent quickly (Figure 5d). The sensor demonstrates a rapid response, with an average response time of 0.21 s and a recovery time of 0.26 s (Figure 5e). The real-time monitoring curves of resistance change for various human body movements, such as bending of the wrist and knee, are also shown in Figure 5f–h. In addition, to monitor subtle signals from the human body, we attached the hydrogel–elastomer stretchable sensor to the volunteer’s throat, and the resistance change exhibited high reproducibility during swallowing (Figure 5i). The results indicate the hydrogel–elastomer stretchable sensor demonstrates excellent potential for monitoring human motions, even subtle signals, in real time.

### 3.6. Applications for 3D Printing

**Figure 6:**
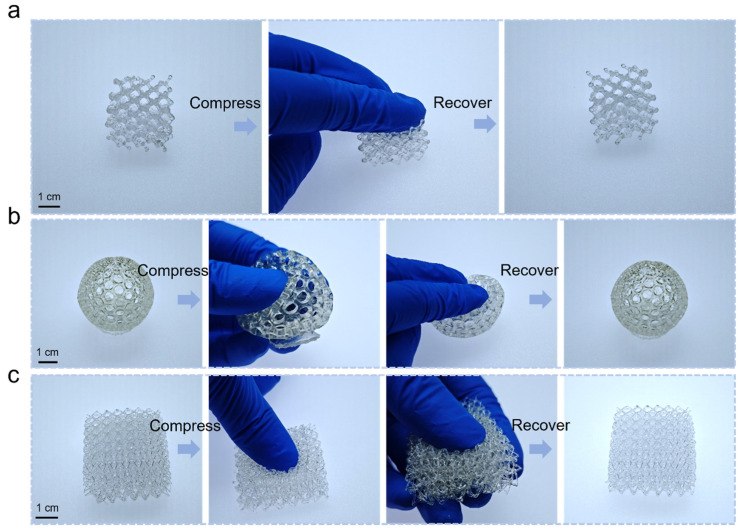
Application of the elastomer for DLP 3D printing. (**a**–**c**) 3D-printed structures can withstand compression and recover to the original shape.

Three-dimensional printing has been widely used in optoelectronics, optical devices, energy, and other fields due to its ability to fabricate smart and functional 3D structures [30,31,32,33]. The most used types include fused deposition modeling (FDM), stereolithography (SLA), digital light processing (DLP), and two-photon lithography 3D printing (TPL) [34,35,36,37]. Among these, the DLP-based photopolymerization method enables the fabrication of complex 3D geometries using spatially UV light to cure liquid resin into 3D objects [38]. However, most liquid resins are rigid, and while some elastic resins have been developed, their stretchability and elasticity are limited [23]. By combining the highly stretchable UV-curable liquid resin we developed with DLP 3D printing, it is possible to fabricate 3D structures and expand the application potential of elastomer. DLP 3D printing (photon-D2) uses digital light projectors to cure UV-curable resin layer by layer. When exposed to the patterned UV light, the resin is solidified and forms a single layer of the desired pattern. This layer-by-layer curing process continues until the entire 3D object is obtained. We define the single layer of printing as 10 μm, exposure time as 10 s, and bottom layer exposure time as 10 s. UVE-P1 liquid resin was used for DLP 3D printing. The preparation step of the liquid resin was shown in the experimental section. The resulting 3D structures, such as lattice structures, exhibit the ability to withstand deformation, such as compression. For example, the printed 3D structures were compressed manually using finger pressure. Once the applied force was removed, the compressed structures recovered to their original shape (Figure 6).

## 4. Conclusions

In conclusion, we introduced a UV-curable elastomer using PUA as an oligomer, HPA as a monomer, PEGDA as a cross-linker, and TPO-L as a photoinitiator. The elastomer was fabricated by UV-curing at a wavelength of 405 nm and exhibited excellent mechanical properties, with a maximum elongation at a break of 2992% and high transparency (94.8% at 550 nm in the visible light region). We demonstrated the fabrication of a robust hydrogel–elastomer stretchable sensor by coating ionic hydrogel onto the surface of the elastomer. The sensor showed high sensitivity and reliability, even under a large strain of 600%. The hydrogel–elastomer stretchable sensor was able to monitor human motions, including finger and wrist-bending, as well as subtle signals, such as swallowing. In addition, the elastomer can be utilized for DLP 3D printing, demonstrating its ability to fabricate complex 3D structures for the potential application of 3D devices. Therefore, this study provides a new elastomer with superior mechanical properties and transparency and demonstrates their potential applications in stretchable and flexible devices and 3D printing.

## Figures and Tables

**Table 1:** Summary of components for the UV-curable liquid resin with different ratios of PUA and HPA.

PUA	HPA	PEGDA	TPO-L	Item
10 g	0 g	0 g	0.3 g	UVE-A1
7.5 g	2.5 g	0 g	0.3 g	UVE-A2
5 g	5 g	0 g	0.3 g	UVE-A3
2.5 g	7.5 g	0 g	0.3 g	UVE-A4

**Table 2:** Summary of components for the UV-curable liquid resin with different PEGDA cross-linkers.

PUA	HPA	PEGDA	TPO-L	Item
5 g	5 g	0 g	0.3 g	UVE-P0
5 g	5 g	PEG200DA 0.05 g	0.3 g	UVE-P1
5 g	5 g	PEG1000DA 0.05 g	0.3 g	UVE-P2

**Table 3:** Comparison of the UV-curable elastomer with previously reported elastomers.

Materials	Transparency	Stretchability	References
PUA-HPA	94.8%	2992%	This work
Epoxy aliphatic acrylate and aliphatic urethane diacrylate	91.6%	1100%	[20]
Polyurethane methacrylate blocking	NA	1605%	[21]
Polyurethane acrylate and isobornyl acrylate	89.4%	414.3%	[24]
Modified TangoPlus	NA	<520%	[23]
Bifunctional urethane acrylate and diluents	NA	<200%	[22]
Poly(mercaptopropylmethylsiloxane-co-dimethylsiloxane)	NA	158%	[25]
Poly(urethane-acrylate) elastomer	90%	600%	[26]
Self-healable polyurethane elastomer	NA	1100%	[27]
Bio-based dimethacrylate compound	NA	66.4%	[28]
Silica-filled silicone elastomer	NA	1400%	[29]

## Data Availability

The original contributions presented in this study are included in this article. Further inquiries can be directed to the corresponding authors.

## References

[1] García Núñez C., Manjakkal L., Dahiya R. (2019). Energy autonomous electronic skin. NPJ Flex. Electron..

[2] Lu N., Kim D.-H. (2014). Flexible and stretchable electronics paving the way for soft robotics. Soft Robot..

[3] Li J., Cao J., Lu B., Gu G. (2023). 3D-printed PEDOT: PSS for soft robotics. Nat. Rev. Mater..

[4] Wang J., Lin M.-F., Park S., Lee P.S. (2018). Deformable conductors for human–machine interface. Mater. Today.

[5] Chen L., Wang Z., Zhan Z., Xie M., Duan G., Cheng P., Chen Y., Duan H. (2021). 3D printed super-anti-freezing self-adhesive human-machine interface. Mater. Today Phys..

[6] Zhuang M., Yin L., Wang Y., Bai Y., Zhan J., Hou C., Yin L., Xu Z., Tan X., Huang Y. (2021). Highly robust and wearable facial expression recognition via deep-learning-assisted, soft epidermal electronics. Research.

[7] Balakrishnan G., Song J., Mou C., Bettinger C.J. (2022). Recent progress in materials chemistry to advance flexible bioelectronics in medicine. Adv. Mater..

[8] Chen L., Liu P., Feng B., Shu Z., Liang H., Chen Y., Dong X., Xie J., Duan H. (2023). Dry-Transferrable Photoresist Enabled Reliable Conformal Patterning for Ultrathin Flexible Electronics. Adv. Mater..

[9] Huang S., Liu Y., Zhao Y., Ren Z., Guo C.F. (2019). Flexible electronics: Stretchable electrodes and their future. Adv. Funct. Mater..

[10] Kim K.K., Ha I., Kim M., Choi J., Won P., Jo S., Ko S.H. (2020). A deep-learned skin sensor decoding the epicentral human motions. Nat. Commun..

[11] Zhang W., Zhang L., Liao Y., Cheng H. (2021). Conformal manufacturing of soft deformable sensors on the curved surface. Int. J. Extrem. Manuf..

[12] Cheng I.-C. (2017). Flexible and printed electronics. Materials for Advanced Packaging.

[13] Raman S., Sankar R. (2022). Intrinsically conducting polymers in flexible and stretchable resistive strain sensors: A review. J. Mater. Sci..

[14] Zhang C., Sun J., Lu Y., Liu J. (2021). Nanocrack-based strain sensors. J. Mater. Chem. C.

[15] Chen F., Gu Y., Cao S., Li Y., Li F., Zhang X., Xu M., Zhang Y. (2017). Low-cost highly sensitive strain sensors for wearable electronics. J. Mater. Chem. C.

[16] Chen J., Zheng J., Gao Q., Zhang J., Zhang J., Omisore O.M., Wang L., Li H. (2018). Polydimethylsiloxane (PDMS)-based flexible resistive strain sensors for wearable applications. Appl. Sci..

[17] Li S., Wu W., Chang Y., Chen W., Liu Y., He Z., Pu Y., Babichuk I.S., Ye T.T., Gao Z. (2024). Flexible strain sensors based on silver nanowires and UV-curable acrylate elastomers for wrist movement monitoring. RSC Appl. Interfaces.

[18] Duan L., D’hooge D.R., Cardon L. (2020). Recent progress on flexible and stretchable piezoresistive strain sensors: From design to application. Prog. Mater. Sci..

[19] Li H., Ma Y., Huang Y. (2021). Material innovation and mechanics design for substrates and encapsulation of flexible electronics: A review. Mater. Horiz..

[20] Patel D.K., Sakhaei A.H., Layani M., Zhang B., Ge Q., Magdassi S. (2017). Highly stretchable and UV curable elastomers for digital light processing based 3D printing. Adv. Mater..

[21] Huang X., Peng S., Zheng L., Zhuo D., Wu L., Weng Z. (2023). 3D Printing of High Viscosity UV-Curable Resin for Highly Stretchable and Resilient Elastomer. Adv. Mater..

[22] Deng Y., Li J., He Z., Hong J., Bao J. (2020). Urethane acrylate-based photosensitive resin for three-dimensional printing of stereolithographic elastomer. J. Appl. Polym. Sci..

[23] Hingorani H., Zhang Y.-F., Zhang B., Serjouei A., Ge Q. (2019). Modified commercial UV curable elastomers for passive 4D printing. Int. J. Smart Nano Mater..

[24] Peng S., Li Y., Wu L., Zhong J., Weng Z., Zheng L., Yang Z., Miao J.T. (2020). 3D Printing Mechanically Robust and Transparent Polyurethane Elastomers for Stretchable Electronic Sensors. ACS Appl. Mater. Interfaces.

[25] Xiang H., Wang X., Ou Z., Lin G., Yin J., Liu Z., Zhang L., Liu X. (2019). UV-curable, 3D printable and biocompatible silicone elastomers. Prog. Org. Coat..

[26] Kim S., Lee J., Han H. (2020). Synthesis of UV curable, highly stretchable, transparent poly (urethane-acrylate) elastomer and applications toward next generation technology. Macromol. Res..

[27] Ye J., Lin G., Lin Z., Deng H., Huang J., Xiang H., Rong M.Z., Zhang M.Q. (2022). UV-Curable Polyurethane Elastomer with UV-Irradiation/Thermo Dual-Activated Self-Healability. Macromol. Mater. Eng..

[28] Fei M., Liu T., Zhao B., Otero A., Chang Y.-C., Zhang J. (2021). From glassy plastic to ductile elastomer: Vegetable oil-based UV-curable vitrimers and their potential use in 3D printing. ACS Appl. Polym. Mater..

[29] Zhao T., Yu R., Li S., Li X., Zhang Y., Yang X., Zhao X., Wang C., Liu Z., Dou R. (2019). Superstretchable and Processable Silicone Elastomers by Digital Light Processing 3D Printing. ACS Appl. Mater. Interfaces.

[30] Chen L., Zhang Y., Ye H., Duan G., Duan H., Ge Q., Wang Z. (2021). Color-Changeable Four-Dimensional Printing Enabled with Ultraviolet-Curable and Thermochromic Shape Memory Polymers. ACS Appl. Mater. Interfaces.

[31] Pan C.-F., Wang H., Wang H., Ruan Q., Wredh S., Ke Y., Chan J., Zhang W., Qiu C.-W., Yang J. (2023). 3D-printed multilayer structures for high–numerical aperture achromatic metalenses. Sci. Adv..

[32] Wang H., Wang H., Zhang W., Yang J. (2020). Toward near-perfect diffractive optical elements via nanoscale 3D printing. ACS Nano.

[33] Wang H., Wang H., Ruan Q., Chan J., Zhang W., Liu H., Rezaei S.D., Trisno J., Qiu C.-W., Gu M. (2023). Coloured vortex beams with incoherent white light illumination. Nat. Nanotechnol..

[34] Zhang W., Wang H., Wang H., Chan J., Liu H., Zhang B., Zhang Y.-F., Agarwal K., Yang X., Ranganath A.S. (2021). Structural multi-colour invisible inks with submicron 4D printing of shape memory polymers. Nat. Commun..

[35] Shahrubudin N., Lee T.C., Ramlan R. (2019). An overview on 3D printing technology: Technological, materials, and applications. Procedia Manuf..

[36] Ge Q., Li Z., Wang Z., Kowsari K., Zhang W., He X., Zhou J., Fang N. (2020). Projection micro stereolithography based 3D printing and its applications. Int. J. Extrem. Manuf..

[37] Wang H., Pan C.-F., Li C., Menghrajani K.S., Schmidt M.A., Li A., Fan F., Zhou Y., Zhang W., Wang H. (2024). Two-photon polymerization lithography for imaging optics. Int. J. Extrem. Manuf..

[38] Guessasma S., Stephant N., Durand S., Belhabib S. (2024). Digital Light Processing Route for 3D Printing of Acrylate-Modified PLA/Lignin Blends: Microstructure and Mechanical Performance. Polymers.

